# Verification of 5-Aminolevurinic Radiodynamic Therapy Using a Murine Melanoma Brain Metastasis Model

**DOI:** 10.3390/ijms20205155

**Published:** 2019-10-17

**Authors:** Junko Takahashi, Shinsuke Nagasawa, Mitsushi J. Ikemoto, Chikara Sato, Mari Sato, Hitoshi Iwahashi

**Affiliations:** 1 Biomedical Research Institute, National Institute of Advanced Industrial Science and Technology (AIST), Ibaraki 305-8566, Japan; m.ikemoto@aist.go.jp (M.J.I.); ti-sato@aist.go.jp (C.S.); ma-satou@aist.go.jp (M.S.); 2 Department of Radiology, Graduate School of Medical Science, Kyoto Prefectural University of Medicine, Kyoto 602-8566, Japan; snaga@koto.kpu-m.ac.jp; 3 Faculty of Applied Biological Sciences, Gifu University, Gifu 501-1193, Japan; h1884@gifu-u.ac.jp

**Keywords:** radiotherapy, radiodynamic therapy, melanoma, brain metastases, 5-aminolevurinic acid, protoporphyrin IX, photodynamic therapy, DNA double-strand break

## Abstract

Melanoma is a highly aggressive cancer with a propensity for brain metastases. These can be treated by radiotherapy, but the radiation-resistant nature of melanoma makes the prognosis for melanoma patients with brain metastases poor. Previously, we demonstrated that treatment of mice with subcutaneous melanoma with 5-aminolevurinic acid (5-ALA) and X-rays in combination, (“radiodynamic therapy (RDT)”), instead of with 5-ALA and laser beams (“photodynamic therapy”), improved tumor suppression in vivo. Here, using the B16-Luc melanoma brain metastasis model, we demonstrate that 5-ALA RDT effectively treats brain metastasis. We also studied how 5-ALA RDT damages cells in vitro using a B16 melanoma culture. Cell culture preincubated with 5-ALA alone increased intracellular photosensitizer protoporphyrin IX. On X-ray irradiation, the cells enhanced their ∙OH radical generation, which subsequently induced γH2AX, a marker of DNA double-strand breaks in their nuclei, but decreased mitochondrial membrane potential. After two days, the cell cycle was arrested. When 5-ALA RDT was applied to the brain melanoma metastasis model in vivo, suppression of tumor growth was indicated. Therapeutic efficacy in melanoma treatment has recently been improved by molecular targeted drugs and immune checkpoint inhibitors. Treatment with these drugs is now expected to be combined with 5-ALA RDT to further improve therapeutic efficacy.

## 1. Introduction

Cutaneous melanoma is a highly aggressive cancer with a propensity for brain metastases. The reported incidence of cerebral metastases varies and depends on whether the source is clinical or autopsy data, and the patient population, among other factors. A study analyzing 700 melanoma patients treated at Roswell Park Memorial Institute from 1972 to 1978 revealed that 125 out of 700 cases (18%) developed brain metastases [1,2]. In contrast, an autopsy study reported melanoma as the most common source of brain metastasis. The incidence of melanoma brain metastasis was recorded as 91%, followed by breast (37%) and lung (34%) cancer [1,3]. In the case of brain metastasis, whole-brain radiotherapy is performed to prevent further metastasis. Palliative treatment, stereotactic radiosurgery (SRS), or stereotactic radiotherapy (SRT) can also be offered. However, melanoma is known as a radiation-resistant cancer due to various mechanisms such as the effect of pigment, free radical scavenging by thiols, or enhanced DNA repair [4], and the prognosis of melanoma patients with brain metastases is still poor [1,5].

5-aminolevulinic acid-mediated photodynamic therapy (5-ALA PDT) has been used to treat precancerous lesions and cancers such as skin cancer [6,7,8]. In particular, 5-ALA ester derivative received marketing authorization for skin cancer in the USA, Europe, and Australia [9]. 5-ALA itself is not a photosensitizer, but it can be converted into a natural photosensitizer, protoporphyrin IX (PpIX), in the mitochondrial intermembrane space. Therefore, exogenous 5-ALA administration leads to PpIX accumulation, causing cellular damage mostly to the mitochondria after light irradiation. Singlet oxygen is the major cytotoxic agent responsible for PDT-induced cellular damage and death.

Previously, we investigated PpIX as a radiosensitizer with oncotropicity. We found that PpIX enhances reactive oxygen species (ROS) generation. Specifically, we used ROS detection reagents to measure the types and amount of ROS generated by X-ray irradiation with ethanol as a ROS quencher in solutions containing different concentrations of PpIX. We estimated that the major ROS present are the hydroxyl radical (OH), superoxide anion (O_2_^−^), and singlet oxygen (^1^O_2_) [10]. Our findings suggest that it is possible to use X-rays as an energy source instead of light. Our in vivo studies using the subcutaneous melanoma model showed that 5-ALA pre-treatment enhanced the effectiveness of X-ray irradiation by acting as a radiomediator that facilitates PpIX accumulation in tumors and enhances ROS production [11,12]. We assessed the combined therapeutic effect of radiodynamic therapy (RDT), using X-rays instead of photodynamic therapy (PDT) laser beams, with 5-ALA as a radiosensitizer. RDT with 5-ALA could have additive/synergistic effects and could be used to target tumors at a depth previously unreachable by laser-based PDT. 5-ALA has also gained clinical approval for fluorescence-guided surgery (FGS) of malignant glioma in many countries [13]. Since 5-ALA is used for the photodynamic diagnosis (PDD) of brain tumors, it is expected to reach brain metastasized melanoma.

Here, we first examined the mechanism of reactive oxygen mediated cellular damage caused by 5-ALA RDT in melanoma, immediately after and 48 h post-irradiation. Next, we verified whether 5-ALA RDT is effective for radiotherapy (RT) on melanoma metastasized to brain using mice with melanoma implanted in the brain—that is, the mouse melanoma brain metastasis model.

## 2. Results

### 2.1. Cellular Response Immediately after Treatment by X-Ray Irradiation with 5-ALA 

To elucidate the mechanism of combined 5-ALA and X-ray treatment, we measured the cellular response immediately after irradiation. 

**Figure 1:**
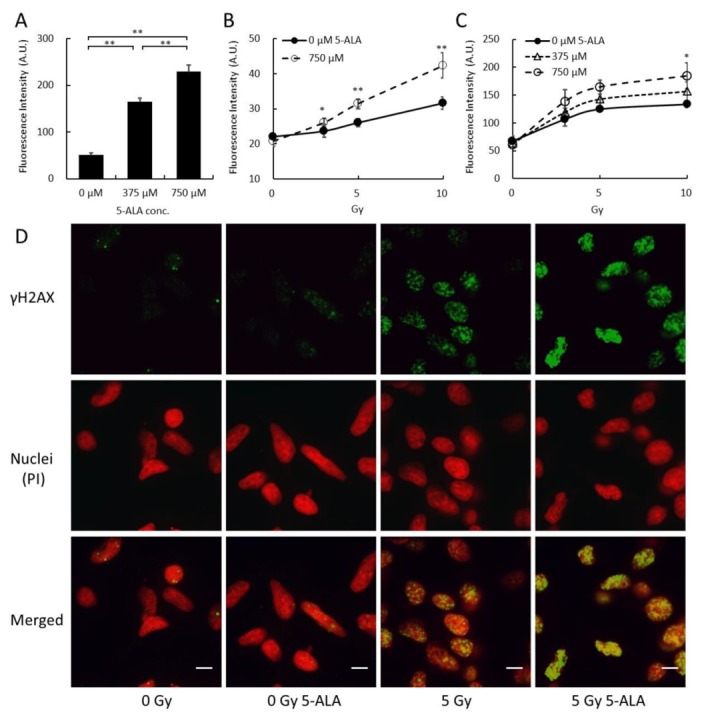
Photosensitizer protoporphyrin IX production and cellular response of 5-ALA-treated melanoma culture immediately after X-ray irradiation. (**A**) Porphyrin levels in B16 mouse melanoma cells in vitro. (**B**) Intracellular ∙OH production levels based on aminophenyl fluorescein (APF) after exposing B16 cells to 5-ALA and X-ray doses. (**C**) γH2AX in cells as a marker of DSBs. (**D**) Subcellular localization of γH2AX (green) and PI-stained nuclei (red) in cells with and without exposure to 5-ALA or X-ray radiation. (**A**) Porphyrin levels were measured using fluorescence intensity at ex/em 405/640 nm. Cells were incubated with different concentrations of 5-ALA for 4 h. (**B**) 5-ALA was added to the culture medium 4 h before irradiation, 10 µM APF was added 30 min before irradiation, and APF levels were measured after irradiation. (**C**) 5-ALA was added 4 h before irradiation. Cells were fixed within 30 min after irradiation and stained for γH2AX immunofluorescence. The cells were analyzed using a flow cytometer. (**D**) Fluorescence in cell culture was imaged using laser confocal microscopy. 5 Gy X-ray radiation induced γH2AX, and 5-ALA pre-incubation in combination with 5 Gy X-ray treatment further enhanced the γH2AX expression. Most of the cells had foci in their nuclei. Data are the means ± SD (*n* = 4). Statistical significance relative to the experiment performed at the same radiation dose is indicated by (* *p* < 0.01, ** *p* < 0.01). Scale bars: 10 µm.

Different concentrations of 5-ALA were added to B16 mouse melanoma cultures 4 h prior to the X-ray irradiation of the cells. The intracellular porphyrin concentration in the culture was determined using fluorescence intensity at an excitation wavelength of 405 nm and an emission wavelength of 635 nm [14]. As the 5-ALA concentration increased, the intracellular PpIX concentration also increased (Figure 1A). 

Next, we used aminophenyl fluorescein (APF) to assess intracellular OH production levels. B16 cells with and without preincubation with 5-ALA were exposed to different X-ray doses. X-ray irradiation in the absence of 5-ALA incubation increased the ∙OH levels in the cells. After 4 h of preincubation with 5-ALA, the OH levels further increased as the radiation doses increased (Figure 1B). 

The histone H2AX is phosphorylated in the vicinity of a DNA double-strand break (DSB) to yield γH2AX, which serves as a marker of DSBs within chromatin. γH2AX is known to increase relative intensity with an increase in the radiation dose [15]. X-ray irradiation without 5-ALA preincubation increased γH2AX levels in B16 cells, while preincubation with 5-ALA further increased the levels of γH2AX, as the radiation doses increased (Figure 1C). Fluorescence microscopy revealed that B16 cells exposed to 5-ALA and 5 Gy X-ray had foci of fluorescence-labeled γH2AX in their nuclei, while cells irradiated by X-ray alone had fewer foci (Figure 1D).

Based on these results, B16 melanoma cells accumulate PpIX in the cells after 5-ALA administration. When the cells are irradiated by X-ray, PpIX enhances ∙OH radical generation, which then induces DNA DSBs.

### 2.2. 5-ALA and X-Ray Irradiation Affect the Cell Cycle Progression In Vitro

**Figure 2:**
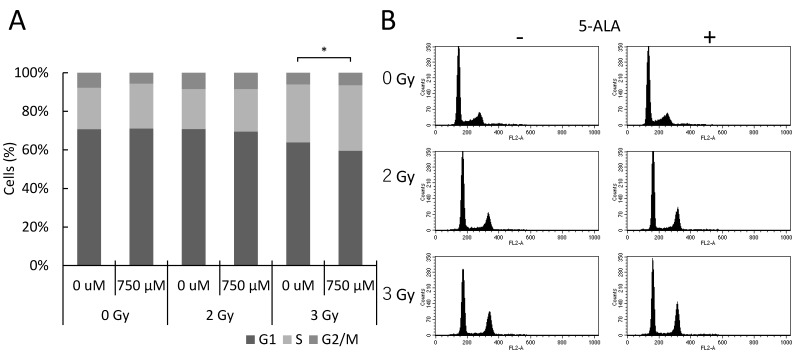
(**A**) Cell cycle distribution 48 h after 2 or 3 Gy irradiation of B16/Bl6 cells in vitro. (**B**) Representative single-parameter histograms of PI fluorescence (DNA content). Cell cycle was interpreted using flow cytometry and PI staining. Data are the means ± SD (*n* = 4). Statistical significance (*p* < 0.05) relative to the experiments performed at the same radiation dose is indicated by (*).

Next, we examined whether the cell cycle of cultured B16 cells was affected by DNA DSBs based on 5-ALA treatment prior to X-ray irradiation in vitro. Forty-eight hours after the irradiation, we performed flow cytometry to analyze propidium iodide (PI)-labeled cells and assessed the DNA content. The results showed that the cell subpopulation in the G1 phase decreased, but that of the S and G2/M phases increased after X-ray irradiation. These effects on the cell cycle were enhanced by 5-ALA treatment (Figure 2A,B). The changes in the cell cycle observed here may be caused by DNA DSBs, which are interpreted to occur immediately after irradiation.

### 2.3. 5-ALA and X-Ray Irradiation Affect the Mitochondrial Membrane Potential In Vitro 

**Figure 3:**
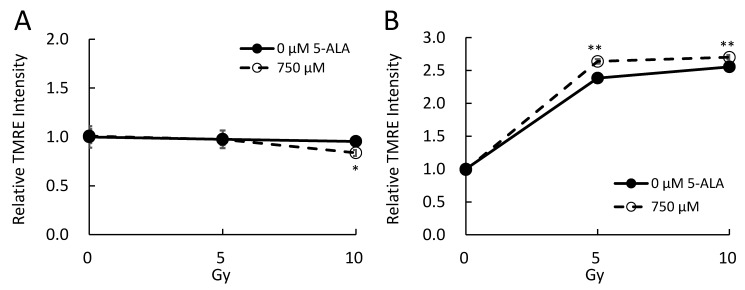
Flow cytometric analysis of mitochondrial membrane potential in B16 cells after ionizing radiation. (**A**) Immediately after irradiation; (**B**) 48 h after irradiation. The mean TMRE fluorescence intensity of each sample was normalized to the control to calculate the relative TMRE intensity. Data are the means ± SD (*n* = 4). Statistical significance relative to the experiment performed without 5-ALA at the same irradiation doses is indicated by * *p* < 0.01, ** *p* < 0.01.

Since PpIX is synthesized in the mitochondria before diffusing and being transported into or outside the cell, a high concentration of PpIX is observed in the mitochondria in the presence of 5-ALA. We examined whether the mitochondrial membrane potential (MMP) of B16 cells was affected by 5-ALA treatment prior to X-ray irradiation in vitro. To measure MMP, we performed flow cytometry to analyze tetramethylrhodamine ethyl ester (TMRE)-labeled cells. The results indicated a significant decrease in MMP immediately after X-ray irradiation; an MMP disruption was observed by X-ray irradiation alone and a greater decrease was seen by the combined treatment with 5-ALA and X-ray (Figure 3A). After 48 h of irradiation, a significant increase in MMP was observed with X-ray alone, and a greater increase was observed following the combined treatment with 5-ALA and X-ray (Figure 3B). The increase in MMP after 48 h of irradiation could be attributable to the increase in both cell size and mitochondria concentration per cell due to cell cycle arrest. It might also be due to the upregulation of the mitochondrial electron transport chain induced by ionizing radiation [16]. 

### 2.4. 5-ALA and X-Ray Irradiation Suppress Tumor Growth in a Brain Metastasis Melanoma Model Mouse

**Figure 4:**
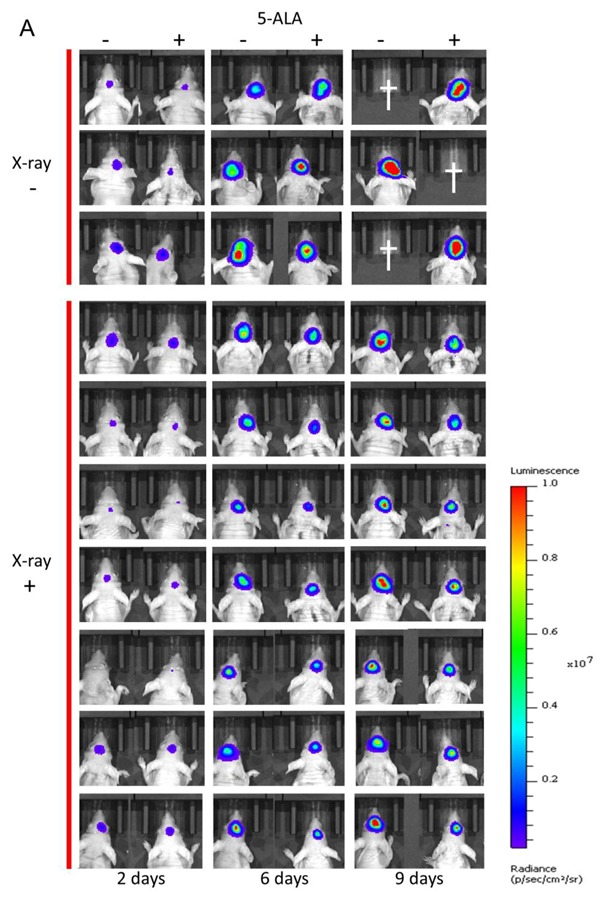

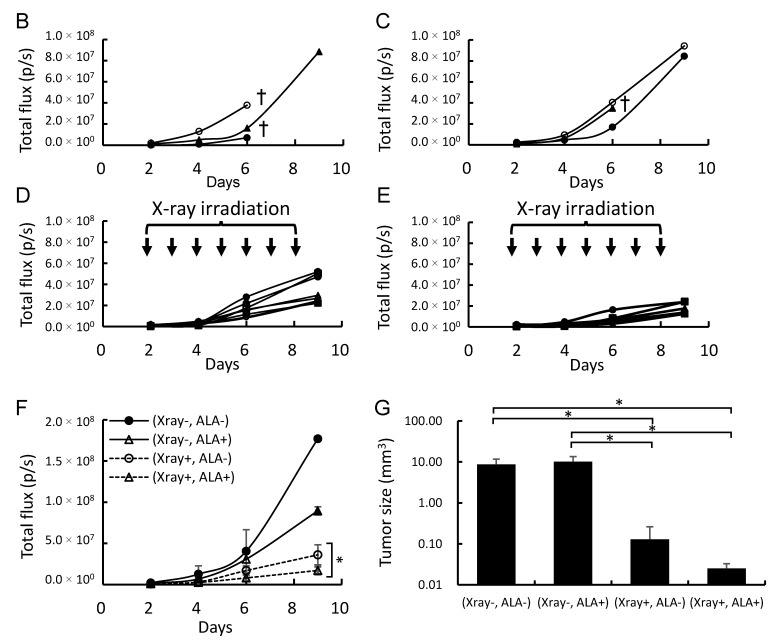
Biophotonic imaging in brain metastatic melanoma model mouse using B16-Luc. (**A**) Cancer growth of each individual, (**B**–**E**) Time course of fluorescence reflecting growth of tumor: (**B**) Control without 5-ALA treatment, (**C**) 5-ALA treatment, (**D**) X-ray treatment of 14 Gy in total (2 Gy/day × 7 days), (**E**) 5-ALA and 14 Gy X-ray treatment (2Gy/day × 7 days). (**F**) Summary of cancer growth of the control and the treated groups. (**G**) Tumor size measured after seven sessions of fractionated irradiation. A 200 mg/kg of 5-ALA was administered intraperitoneally 4 h before X-ray irradiation every day. Daggers indicate the death of the animal. Statistical significance (*p* < 0.05) relative to the experiment performed without 5-ALA at the same irradiation doses is indicated by (*).

The mouse B16-Luc melanoma brain metastasis model was used to evaluate the effect of combined 5-ALA and ionizing radiation treatment on tumor suppression in vivo. Two days after B16-Luc cell transplantation, the mouse brain was irradiated with 2 Gy daily for seven days, or 14 Gy in total. 5-ALA (200 mg/kg) was administered intraperitoneally every day 4 h before X-ray irradiation. The dose and timing of 5-ALA administration were based on the clinical conditions of PDD. Porphyrin accumulation in implanted B16-Luc tumors 4 h after 5-ALA administration was 5.3 ± 1.4 pM/mg protein, which is 26 times higher than that without administration. Tumor growth was fluorescently monitored two, four, six, and nine days after cell transplantation. The tumor growth of the non-irradiation group was very rapid. However, X-ray irradiation suppressed the tumor growth, and combined treatment with 5-ALA and X-rays further suppressed the tumor growth, as revealed by bioluminescence imaging analysis (Figure 4A–F). Similar results were obtained when the tumor size was anatomically measured after seven fractional irradiations (Figure 4G).

## 3. Discussion

Melanoma is the primary human cancer with the highest propensity to metastasize to the brain. The prognosis of patients with melanoma brain metastases is extremely poor. The only effective treatment until recently has been surgical extirpation, when feasible, due to melanoma’s radioresistance and poor response to existing chemotherapeutic regimes [17]. Melanoma’s radioresistance is thought to be caused by various mechanisms, including pigmentation, free radical scavenging by thiols, and enhanced DNA repair [4]. Śniegocka et al. have demonstrated three main mechanisms of cellular radioprotection of melanin [18]. Firstly, melanin can absorb ionizing irradiation due to the broadened spectrum of absorption and efficient internal conversion of the absorbed energy. Secondly, melanin recombines with water radiolysis products and other melanotic cell compounds. Finally, melanin consumes and adsorbs oxygen, and the process of melanin synthesis consumes a significant amount of the cosubstrate oxygen (e.g., for tyrosinase, phenylalanine hydroxylase, etc.). Sparsa et al. examined the effect of melanin pigment on PDT using two strains with or without melanin: B16F10 and B16Gf4 cells [19]. Cell death corresponded to p53-dependent apoptotic signaling in pigmented B16F10 cells, but an autophagic response leading to caspase-independent death was detected in nonpigmented B16G4F cells. Therefore, the PDT-induced cell death pathway appears to correlate with melanin synthesis capacity in melanoma cells. The resistance of melanoma cells to RT may also be related to constitutive MAPK pathway activation, or the inactivation of p53 observed in about 90% of melanomas [20,21]. Krayem et al. showed that p53 activator (PRIMA-1Met) and BRAF inhibitor (Vemurafenib) both improve the radiosensitizing effect of mutated BRAF melanoma cells [4].

Here, we used a strain with melanin, so the experimental design ensured that the X-ray irradiation effect would be difficult to manifest. In our previous study, no induction of the p53 pathway was observed after combined PpIX or 5-ALA administration and irradiation in a strain with melanin (B16-BL6 cells) [11] or a strain without melanin (HeLa cells) [22], but the RT effect improved. These results suggest that the mechanism of RDT on cells differs from PDT, and p53 pathway activation may improve RDT.

Significant therapeutic advances have occurred in the field of metastatic melanoma in recent years. The advent of BRAF inhibitors, immune checkpoint inhibitors (ICIs), and their combination/sequence has revolutionized clinical practice, leading to significant survival prolongation compared to older cytotoxic strategies. Recently, preclinical and limited clinical evidence supported the existence of a synergistic effect between brain RT and immunotherapy in metastatic melanoma [23]. Chicas-Sett et al. reviewed the combination of RT and the immune checkpoint inhibitor (ipilimumab, anti-CTLA-4 antibody), which induces clinically relevant radiation-induced abscopal effects in metastatic melanoma patients [5]. Their early clinical outcome reports suggest that the combination of ipilimumab and RT may improve survival in metastatic melanoma patients.

For RDT, Yamamoto et al. showed immunohistochemical analysis of a rat glioma subcutaneous model to reveal that numerous ionized calcium-binding adapter molecule 1 (Iba1)-positive macrophages gathered at the surface and within the subcutaneous tumors after multidose ionizing irradiation in combination with 5-ALA administration [24].

Regarding RT dosage in melanoma, a multi-institutional retrospective study was performed in northern Japan to analyze the outcome of external RT as the definitive treatment modality for localized mucosal melanoma of the head and neck. As a result, RT at a dose of 3 Gy or more per fraction could effectively gain local control in patients with localized mucosal melanoma of the head and neck, and allowed for better survival, especially in younger patients [25]. In Chandra’s systematic review, the case reports and preclinical data of melanoma patients suggest that RT and immunotherapy (ipilimumab) may synergize to generate “abscopal” responses outside the radiation field, and multiple fraction radiation regimens at a dose of 3 Gy or higher were associated with a more favorable response [26]. Dewan et al. also tested the hypothesis that the type of dose fractionation regimen determines the ability of RT to synergize with the anti-CTLA-4 antibody [27]. The abscopal effect occurred only in mice treated with the combination of fractionated RT, and hypofraction conditions were the most effective.

Here, the 5 Gy X-ray irradiation induced the expression of γH2AX, and 5-ALA pretreatment further enhanced the γH2AX expression (Figure 1C,D), reflecting critical cellular response to DNA damage. In our previous in vivo studies, the effect of 20 Gy (2 Gy, 10 fractions) combined with 5-ALA had an effect equivalent to 30 Gy (3 Gy, 10 fractions) [12]. The combined use of 5-ALA and X-rays may induce the same effects as a higher dose, causing an immune response that destroys tumor cells. Thus, further investigation of the RDT-induced immune response is necessary. The effect of RDT appears to be strong in mitochondria since PpIX is synthesized in the mitochondrial intermembrane space. Even though MMP was examined in this study, it is necessary to further investigate the effect on mitochondrial function and morphology [28,29].

The advantage of 5-ALA is a huge accumulation of data thanks to previous PDT and PDD studies. In particular, PpIX accumulation in tumors is due to cancer’s common property [6,7,8,9]. The reason for the advanced clinical use of 5-ALA is that PpIX accumulation occurred in cancer in response to an oral dose of heme precursor 5-ALA [30]. Furthermore, it was not phototoxic compared to other photosensitizers. The ability to be administered orally and the absence of phototoxicity are two critical advantages to RDT’s use as a radiosensitizer, especially with the continued use of daily fractional radiation. Thus, the combined use of 5-ALA for melanoma RT is expected to improve the therapeutic effect significantly. As the therapeutic results of melanoma have been improved by the combined use of molecular targeted drugs or ICIs in recent years, treatment with these drugs combined with 5-ALA RDT is strongly expected to further improve the therapeutic effects.

## 4. Materials and Methods

### 4.1. Chemicals

5-ALA hydrochloride, RPMI 1680 medium, penicillin, streptomycin, fetal bovine serum (FBS), phosphate-buffered saline (PBS), NaOH, N,N-dimethylformamide, isopropanol, propidium iodide (PI), RNase, and D-luciferin were purchased from Wako Chemicals (Osaka, Japan). A modified Lowry protein assay kit was purchased from Pierce (Rockford, IL, USA). Aminophenyl fluorescein (APF) was purchased from Goryo Chemical (Sapporo, Japan). A DNA damage detection kit (containing γH2AX monoclonal antibody and secondary antibody) was purchased from Dojindo Laboratories (Kumamoto, Japan). A MitoPT^®^ TMRE Mitochondrial Depolarization Assay Kit was purchased from ImmunoChemistry Technology (Bloomington, MN, USA).

### 4.2. Cell Culture

B16F10 melanoma cells (B16-Luc) were supplied by Sanford Burnham Prebys Medical Discovery Institute (La Jolla, CA, USA). B16-Luc cells were cultured in RPMI 1640 containing 10% FBS in a 5% CO_2_ humidified incubator at 37 °C. The medium was supplemented with 100 units/mL penicillin and 100 μg/mL streptomycin.

### 4.3. X-Ray Irradiation Conditions

X-ray irradiation was carried out in a Faxitron CP-160 irradiator (Faxitron X-ray Corporation, Wheeling, IL, USA) with X-ray energy outputs of 160 kV. A free-air ionization chamber (RAMTEC1500-DC300, ToyoMedic Ltd., Tokyo, Japan) was used for dose rate measurement. The resulting dose rate was 1.0 Gy/min at the sample stage.

### 4.4. Determination of PpIX Concentration in Cells and Tissue

The relative concentration of PpIX was measured in cultured cells. B16 cells were cultivated in 96-well plates until confluent. 5-ALA was added to the culture medium at a final concentration of 0, 375, or 750 μM and incubated for 4 h. After washing with PBS, the relative concentration of PpIX was determined using a fluorescence multiwell plate reader (Infinite M200, TECAN, Kanagawa, Japan) at excitation/emission and λ = 405 nm/635 nm. PpIX in tissue samples was isolated in 50 μL 0.1 M NaOH and homogenized on ice with Powermasher II (Assist, Tokyo, Japan). An aliquot (10 μL) of the NaOH-treated sample was withdrawn and used for a protein concentration assay (modified Lowry protein assay kit), while the remaining 40 μL of the NaOH-treated sample were denatured by the addition of 150 μL of N,N-dimethylformamide:isopropanol (100:1, *v*/*v*) solution. The prepared sample, after overnight storage, was centrifuged at 12,000 rpm for 10 min. The porphyrin concentration was determined by spectrophotometry at the Soret maximum (405 nm), and the fluorescence using an excitation wavelength of 405 nm and an emission wavelength of 635 nm [14].

### 4.5. Measurement of Intracellular ROS

Hydroxyl radical (OH) generation was measured using APF as a detection agent [31]. B16 cells were cultivated in 25-cm^2^ tissue culture flasks to confluency. 5-ALA was added 4 h before X-ray irradiation in 5 mL of culture medium at a final concentration of 0, 375, or 750 μM. APF was added at a final concentration of 10 μM. The plates were incubated in the dark for 30 min at 37 °C and irradiated with a dose of 0, 3, 5, or 10 Gy. The cells were collected after irradiation. Cells were diluted to 10^6^ cells/mL in PBS and transferred to a black well plate. Fluorescence was measured using a fluorescence multiwell plate reader (Infinite M200) at excitation/emission and λ = 480 nm/520 nm for APF.

### 4.6. γH2AX Detection

For the measurement of γH2AX using a flow cytometer, B16 cells were cultured in six-well plates until they became confluent, and radiated by X-rays. Within 30 min of the irradiation, the cells were collected and fixed. The cells were then permeabilized with 0.25% Triton X-100/ PBS and blocked with 1% BSA. The cells were stained with anti-γH2AX antibody and further with a green fluorescence-labeled secondary antibody. The cells were then analyzed using a FACSCalibur flow cytometer (BD BioSciences, San Diego, CA, USA). For immunofluorescence microscopy, cells were separately grown on glass chamber slides to observe the distribution of γH2AX. The cells were fixed within 30 min of irradiation. The cells were stained with γH2AX using a secondary antibody tagged with green fluorescein, while DNA was counter-stained with PI (0.5 μg/mL). Cells were treated with RNase (0.25 mg/mL) prior to staining with PI. Cells were finally imaged using a laser confocal microscope, a BX system (Olympus Inc., Tokyo, Japan) with a confocal unit CSU22 (Yokogawa Inc., Tokyo, Japan), with a 60× water-immersion objective lens (NA: 1.1, Olympus Inc., Tokyo, Japan).

### 4.7. Cell Cycle Assay

For the cell cycle assay, 750 μM 5-ALA was added into sub-confluent B16-Luc cells in six-well plates 4 h before X-ray irradiation. After X-ray irradiation with a dose of 2 or 3 Gy, the cells were washed twice with PBS and incubated with fresh medium. The cells were collected 48 h after irradiation, fixed in 70% ethanol, and stored at −20 °C. The cells were washed twice with PBS, resuspended in PBS containing 0.25 mg/mL RNase, incubated at 37 °C for 30 min, and stained with PI (50 μg/mL) at 4 °C for 30 min. The DNA content of the cells was analyzed using a FACSCalibur flow cytometer. The percentages of cells in different phases of the cell cycle were calculated using FlowJo software (Treestar, Inc., San Carlos, CA, USA) with the Watson pragmatic cell cycle modeling algorithm.

### 4.8. Mitochondrial Membrane Potential

MMP was quantified by staining cells with the potentiometric fluorescent tetramethylrhodamine ethyl ester (TMRE) dye. To assess MMP, 750 μM 5-ALA was added into subconfluent B16 cells in 25-sq cm tissue culture flasks 4 h before X-ray irradiation. After X-ray irradiation, the cells were washed twice with PBS and incubated with fresh medium. After 30 min or 48 h of irradiation, the cells were incubated in RPMI 1640/10% FBS containing 20 nM TMRE for 30 min at 37 °C. The cells were trypsinized and washed twice with PBS. After the cells were resuspended in serum-free RPMI 1640 medium, they were analyzed using a FACSCalibur flow cytometer. The mean TMRE fluorescence intensity of each sample was normalized to the control sample to calculate the relative TMRE intensity. Carbonyl cyanide m-chloro phenylhydrazine (CCCP) at a concentration of 10 µM was used as a positive control.

### 4.9. Intracranial Implantation of B16-Luc Melanoma Cells and X-Ray Irradiation in Mice

A mouse melanoma B16 subline, B16-Luc stably integrated with a luciferase reporter gene, was used to establish an intracranial xenograft as a brain metastatic melanoma model. Six-week-old female nude mice (Charles River Laboratories Japan, Inc. Yokohama, Japan) were anesthetized and 10,000 B16-Luc cells were stereotactically injected in the corpus callosum (1 mm anterior to bregma, −1 mm lateral and −3 mm in deep of the cortex surface) using a stereotactic instrument (Narishige, Tokyo, Japan) as previously described [32]. Animals were observed until full recovery. A total of 20 mice were injected for B16-Luc. Two days after cell transplantation, tumor growth was monitored and measured using in vivo bioluminescence imaging with the IVIS 100 system (Caliper Life Sciences, Alameda, CA, USA) as previously described [12]. They were divided into four groups to ensure fluorescence uniformity: (1) control group (*n* = 3), (2) 5-ALA treatment (*n* = 3), (3) X-ray treatment (*n* = 7), and (4) 5-ALA and X-ray treatment (*n* = 7). Two days after cell injection, the mice in the X-ray and 5-ALA and X-ray treatment groups were irradiated with 2 Gy daily q.d. (quaque die) × 7 days for a total dose of 14 Gy. For X-ray irradiation, a mouse was tightly held in a plastic holder with an opening above the tumor area. The collimated X-ray beam irradiated a 20 × 20 mm area of the brain at the tumor site, which was large enough to cover the entire tumor. Mice in the 5-ALA and X-ray treatment group were intraperitoneally administered 5-ALA diluted in PBS at 200 mg/kg body weight 4 h before X-ray irradiation. Mice in the X-ray treatment group were intraperitoneally administered PBS 4 h before X-ray irradiation. The mice in the 5-ALA treatment group and control group received 5-ALA or PBS at the same time. The body weight and intracranial mouse tumors were recorded two, four, six, and nine days after cell transplantation. After the last seven sessions of X-ray irradiation, the mice were sacrificed. The tumor size in the brain was measured from the photograph with a caliper, using the following formula: tumor size (mm^3^) = length × width^2^ × 0.5. All experimental protocols were approved by the Committee for the Care and Use of Experimental Animals at AIST (Permit Number: 2019–097, 2019/04/01).

### 4.10. In Vivo Imaging of Intracranial Tumors

Intracranial tumor growth was quantified using biophotonic imaging with an IVIS 100 system. Mice were administered a 100 μL intraperitoneal injection of 30 mg/mL D-luciferin suspended in PBS 10 min before imaging as a substrate for the luciferase enzyme. Anesthesia was induced with isoflurane gas prior to imaging by placing the mice in the chamber of an XGI-8 vaporizer. Anesthesia was sustained by inhalation via nose cones inside the imaging chamber. Images were captured and quantified using Living Image 4.4 software (Caliper Life Sciences, Hopkinton, MA, USA) based on equivalent regions of interest over the head. Image intensities were expressed as total flux (p/s).

### 4.11. Statistics

PpIX, cell cycle, and tumor growth were analyzed using one-way factorial ANOVA followed by the Tukey‒Kramer multiple comparisons test. The Games‒Howell post hoc test was used in cases when the variance was not homogenous. Intracellular ROS and MMP were analyzed using the two-tailed Student’s *t*-test. Differences were considered statistically significant at *p* < 0.05.

## 5. Conclusions

This study shows that the intracellular mechanism of RDT in melanoma is as follows. (1) 5-ALA induces the accumulation of PpIX in cancer cells. (2) PpIX enhances ∙OH production by X-ray irradiation. (3)∙OH induces DNA DSBs, resulting in cell cycle arrest. Moreover, the combined treatment of 5-ALA and X-rays was effective in a melanoma mouse brain metastasis model. Thus, RDT is expected to enhance RT of melanoma brain metastases.

## Abbreviations

5-ALA	5-aminolevurinic acid
DSB	double-strand break
MMP	mitochondrial membrane potential
PDD	photodynamic diagnosis
PDT	photodynamic therapy
PpIX	protoporphyrin IX
RDT	radiodynamic therapy
ROS	reactive oxygen species
RT	radiation therapy
